# Supplementary material for: Tailoring polymer electrolyte ionic conductivity for production of low- temperature operating quasi-all-solid-state lithium metal batteries
Source: Nat Commun. 2023 Jan 30;14:482. doi: 10.1038/s41467-023-35857-x (PMC9886912; doi:10.1038/s41467-023-35857-x)
Supplement: Supplementary file 1 — Supplementary Information [file 41467_2023_35857_MOESM1_ESM.pdf]

Supplementary Information

Tailoring polymer electrolyte ionic conductivity for production of low-temperature operating quasi-all-solid-state lithium metal batteries

Zhuo Li[1,4], Rui Yu[1,4], Suting Weng[2], Qinghua Zhang[2], Xuefeng Wang[2,3,], Xin Guo[1,*]*

[1] School of Materials Science and Engineering, State Key Laboratory of Material Processing and Die & Mould Technology, Huazhong University of Science and Technology, Wuhan 430074, P. R. China.
[2] Laboratory of Advanced Materials and Electron Microscopy, Institute of Physics, Chinese Academy of Science, Beijing 100190, P. R. China.
[3] Tianmu Lake Institute of Advanced Energy Storage Technologies Co. Ltd., Liyang, Jiangsu 213300, P. R. China
[4] These authors contributed equally: Z. Li, R. Yu

* E-mail: xguo@hust.edu.cn (X. Guo), wxf@iphy.ac.cn (X. Wang)

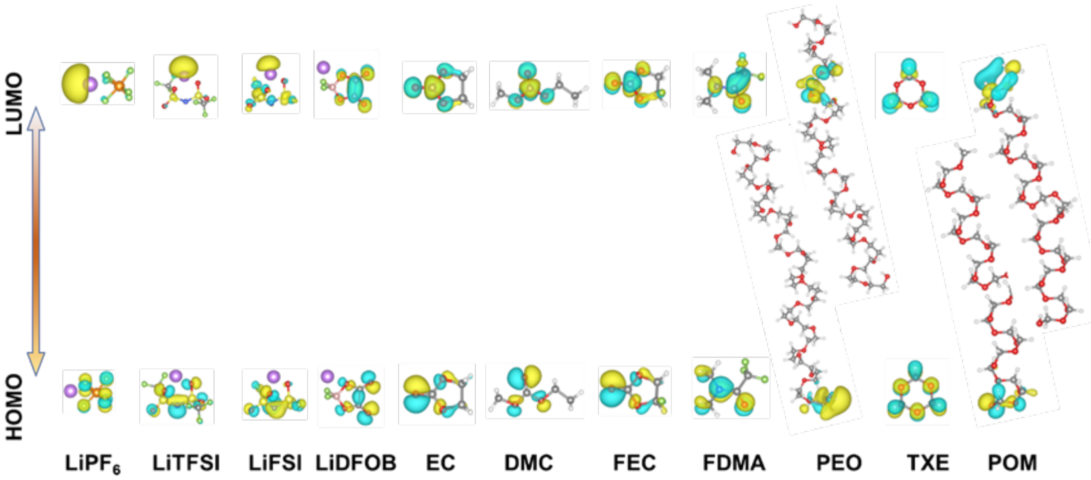

Supplementary Fig. 1 HOMO and LUMO orbitals of commonly used solvents, Li salts and polymers.

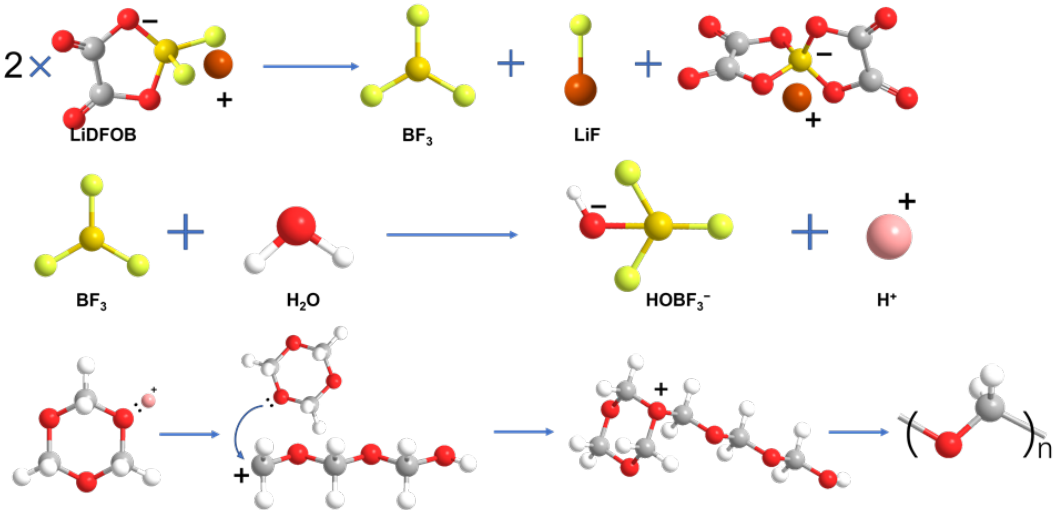

Supplementary Fig. 2 Polymerization mechanism of TXE monomers induced by LiDFOB.

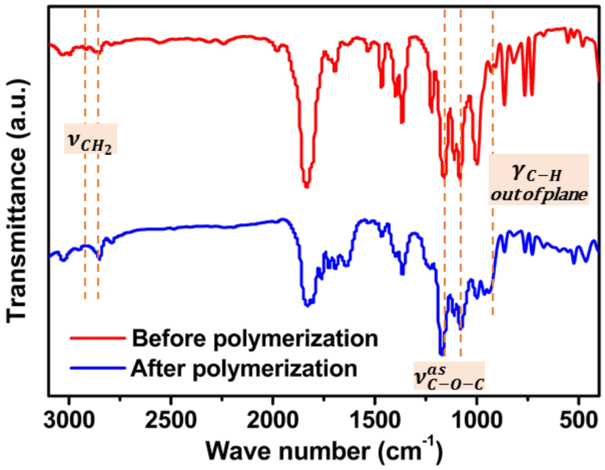

Supplementary Fig. 3 Structure characterization of the polymer electrolyte by FTIR before and after polymerization.

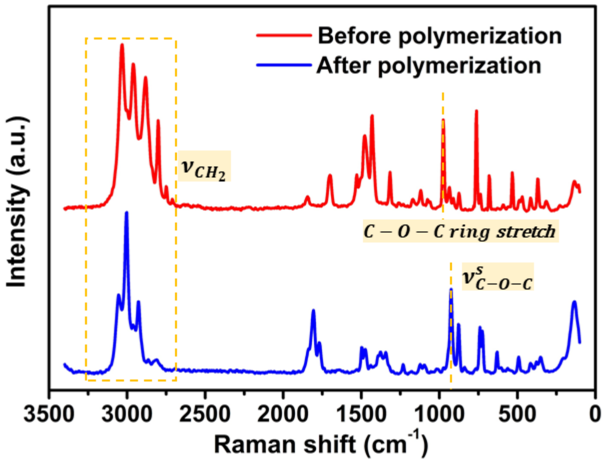

Supplementary Fig. 4 Raman spectra of the polymer electrolyte before and after polymerization.

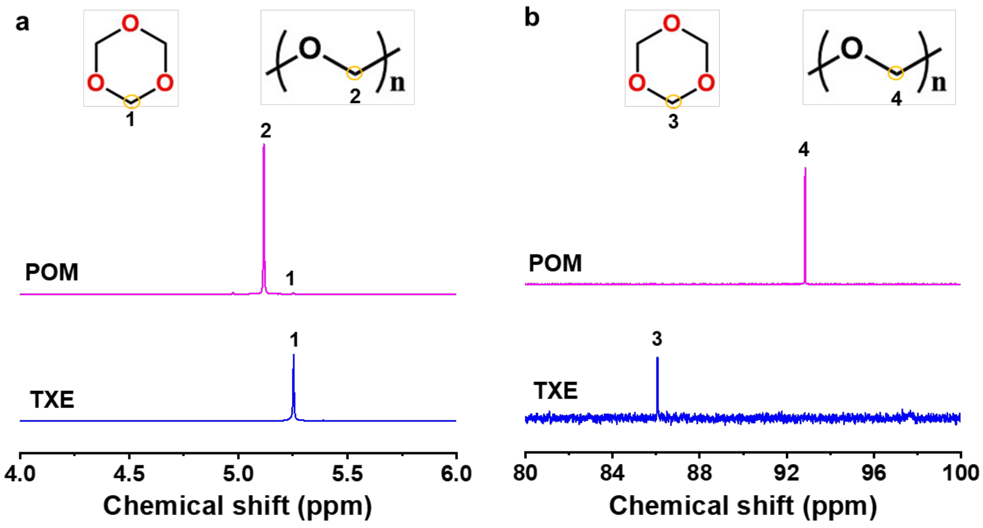

Supplementary Fig. 5 NMR spectra of TXE and POM: **a**. hydrogen, **b**. carbon.

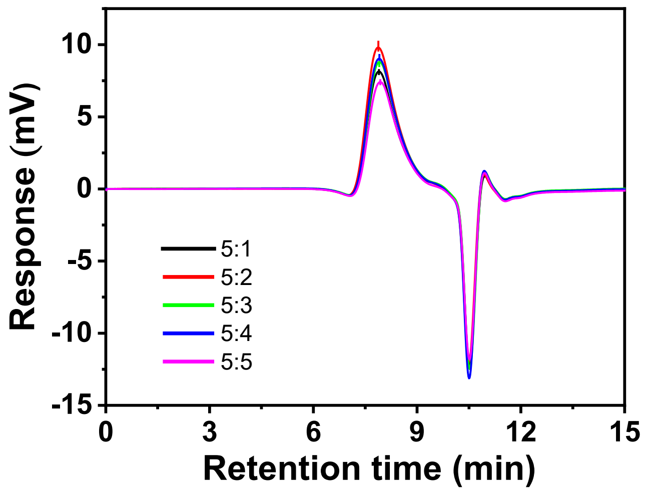

Supplementary Fig. 6 Gel permeation chromatography (GPC) test results of prepared electrolytes with different TXE-FDMA ratios.

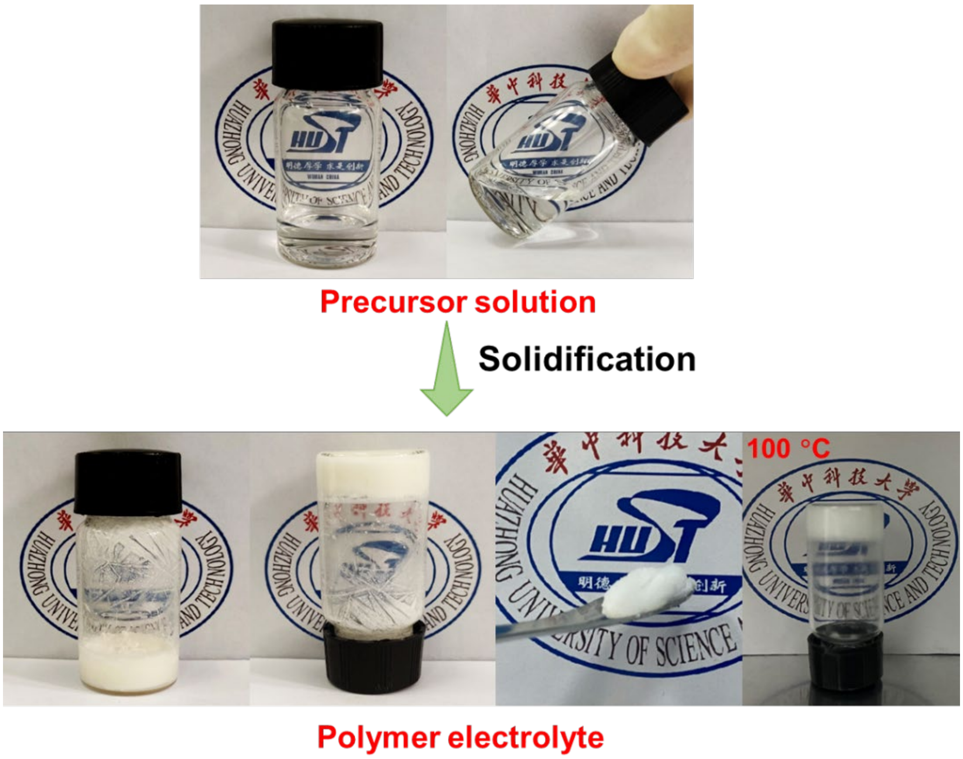

Supplementary Fig. 7 Optical images of the precursor and the polymer electrolyte.

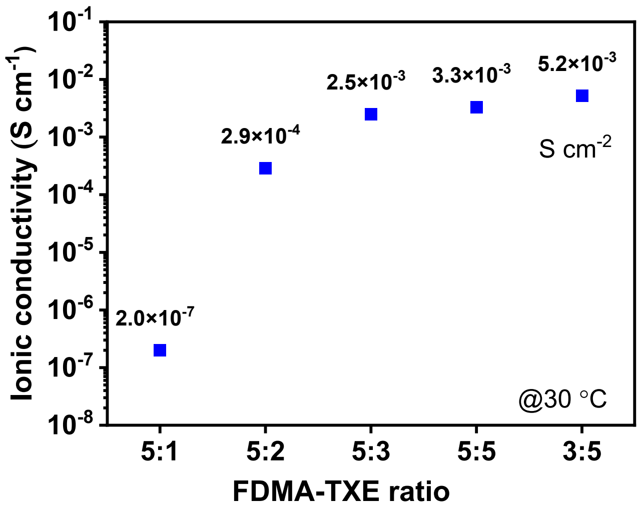

Supplementary Fig. 8 Ionic conductivities of polymer electrolytes at 30 °C with various ratios of TXE and FDMA. The ionic conductivities of the electrolyte samples were calculated from EIS results. A homemade bottle cell with two stainless steel electrodes inside was used for EIS measurements, which were carried out at an alternating potential amplitude of 10 mV in a frequency range of 5 MHz to 1 Hz and with 10 points per decade.

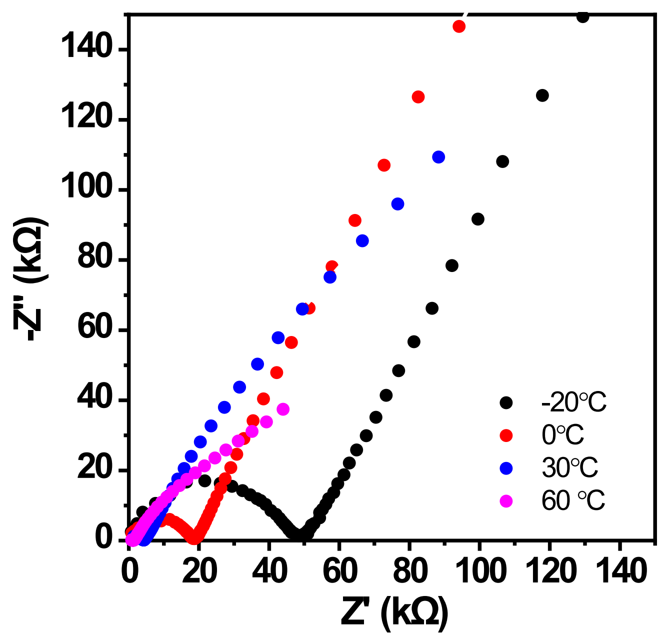

Supplementary Fig. 9 Electrochemical impedance spectroscopy (EIS) measurements of the polymer electrolyte at various temperatures. The EIS measurements were carried out by using a stainless steel||stainless steel blocking cell at an alternating potential amplitude of 10 mV in a frequency range of 5 MHz to 1 Hz and with 10 points per decade.

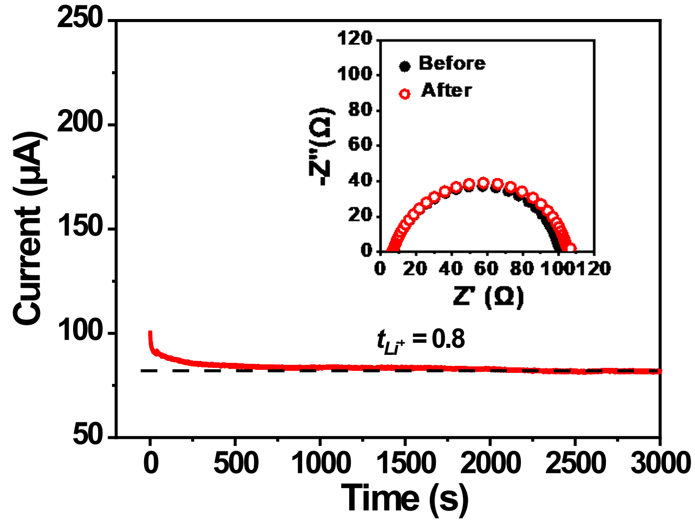

Supplementary Fig. 10 Chronoamperometry profile of the polymer electrolyte in the Li||Li coin cell at 30 °C, inset is the Nyquist plots before and after chronoamperometry. The chronoamperometry and EIS (at an alternating potential amplitude of 10 mV in a frequency range of 5 MHz to 1 Hz and with 10 points per decade) were carried out by using the Li||Li coin cell.

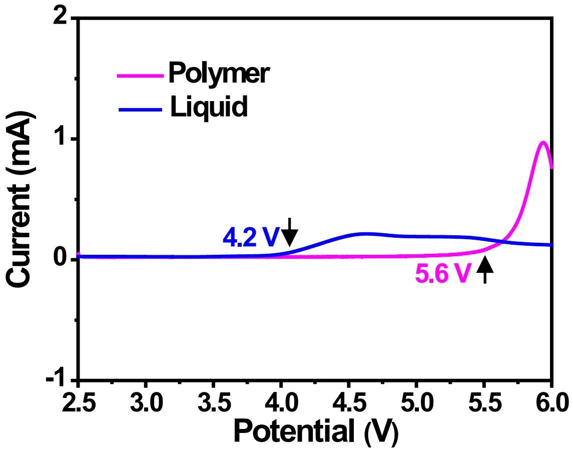

Supplementary Fig. 11 Positive linear sweep voltammogram (LSV) of Li||Pt coin cell (at 30 °C) to gauge the oxidation stability of both electrolytes.

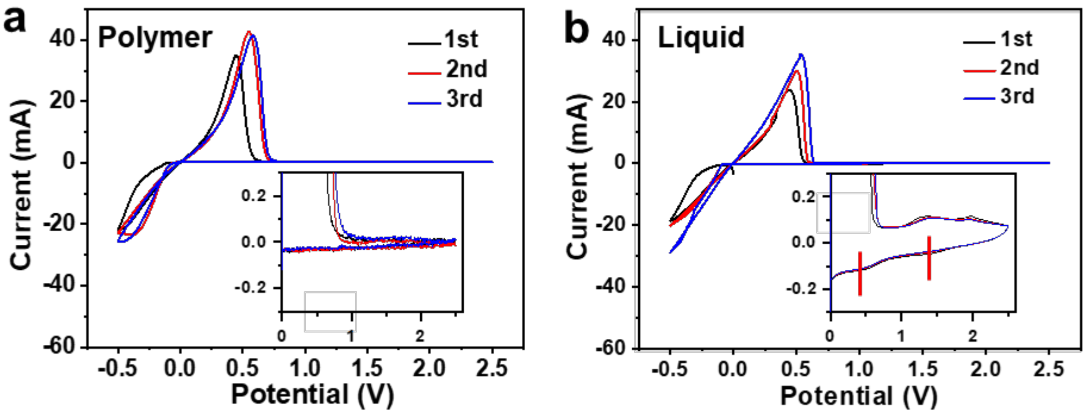

Supplementary Fig. 12 Cyclic voltammetry (CV) curves of both electrolytes in Li||Pt coin cells at 30 °C.

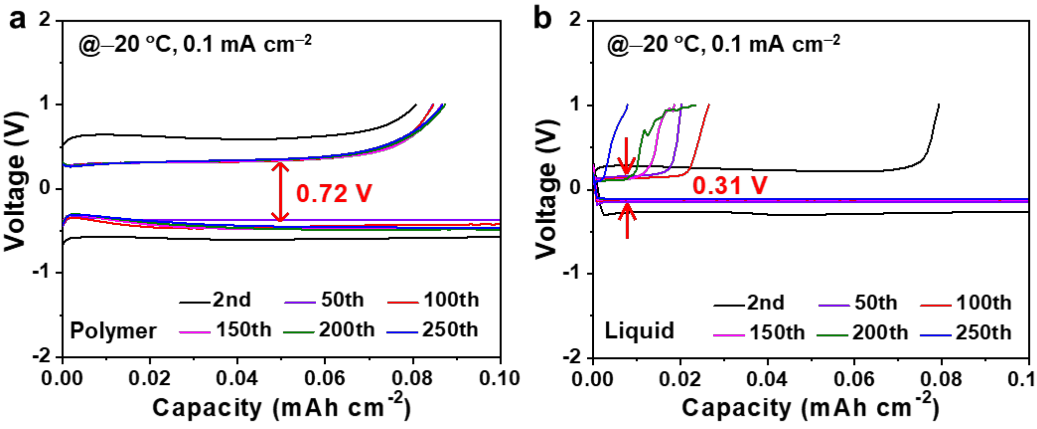

Supplementary Fig. 13 Voltage profiles for Li plating/stripping on Cu working electrode for both electrolytes cycled in Li||Cu coin cells: **a**. polymer electrolyte, **b**. liquid electrolyte.

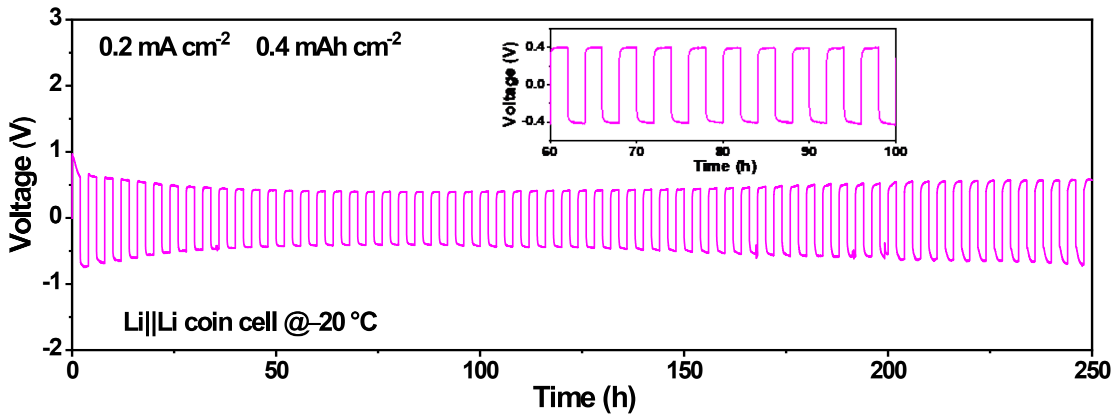

Supplementary Fig. 14 Galvanostatic cycling of the polymer electrolyte in symmetric Li||Li coin cell with 50 μm Li foil at a high capacity of 0.4 mAh cm^{-2} (0.2 mA cm^{-2}) and at −20 °C.

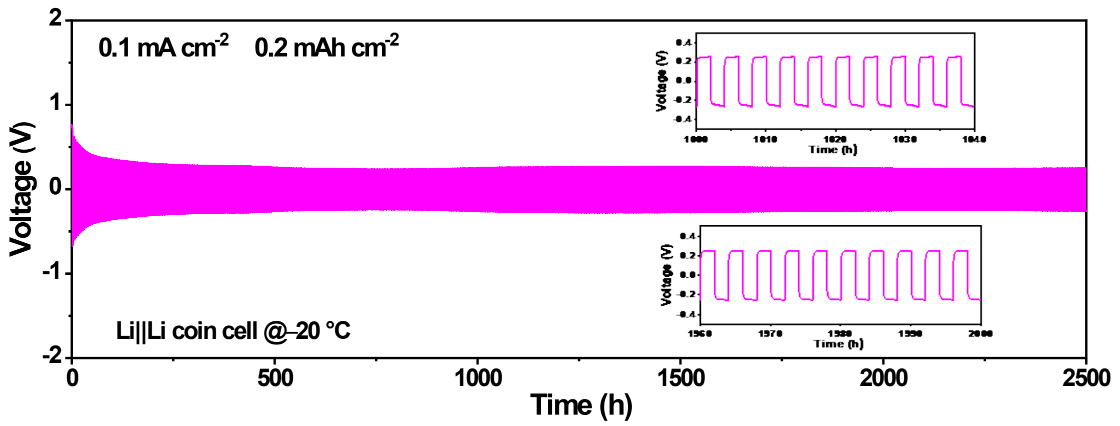

Supplementary Fig. 15 Galvanostatic cycling of the polymer electrolyte in symmetric Li||Li coin cell using 250 μm Li foil at −20 °C.

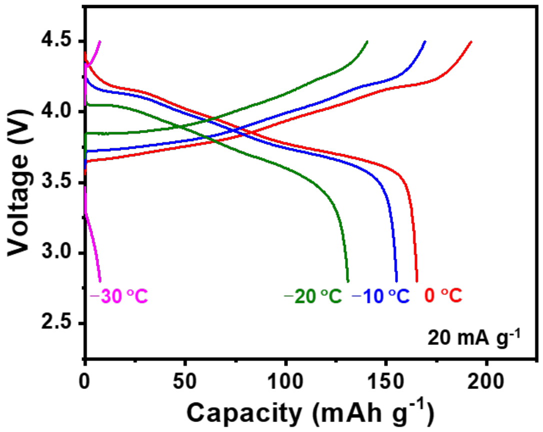

Supplementary Fig. 16 Charge-discharge profiles of the liquid electrolyte in $Li||LiNi_{0.8}Co_{0.1}Mn_{0.1}O_2$ (NCM811) coin cell at different temperatures. The reported cycles are the second cycle at each temperature.

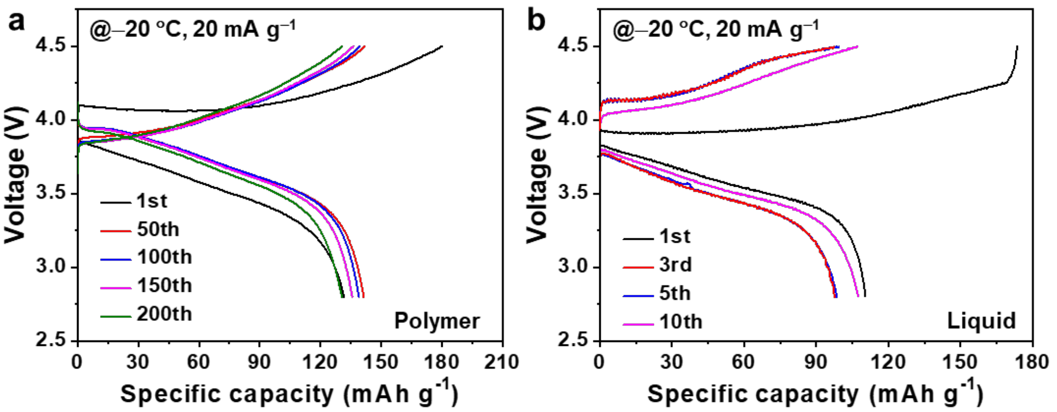

Supplementary Fig. 17. Typical charge-discharge voltage profiles of both electrolytes in Li||NCM811 coin cells at –20 °C and 20 mA g^{-1}: **a**. liquid electrolyte, **b**. polymer electrolyte.

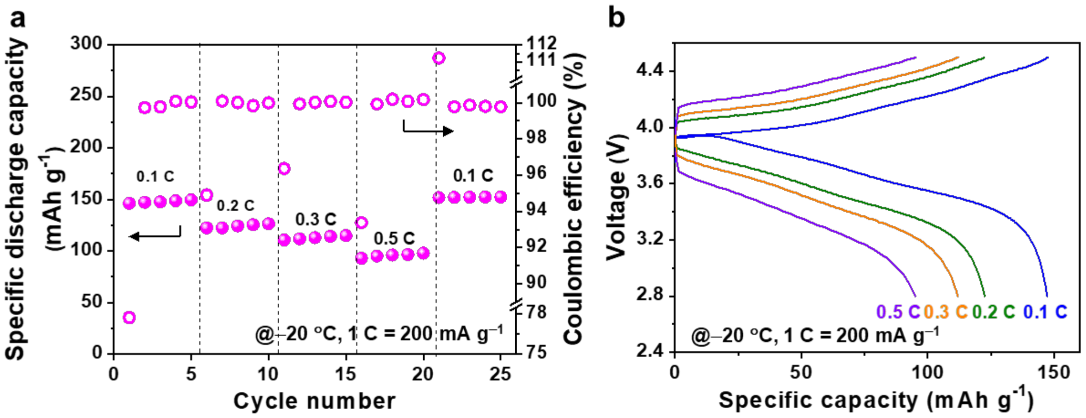

Supplementary Fig. 18 a. Rate performance of the Li||NCM811 coin cell with the designed electrolyte at –20 °C, and **b**. corresponding charge-discharge voltage profiles at different C-rates. The reported cycles are the second cycle at each C-rate.

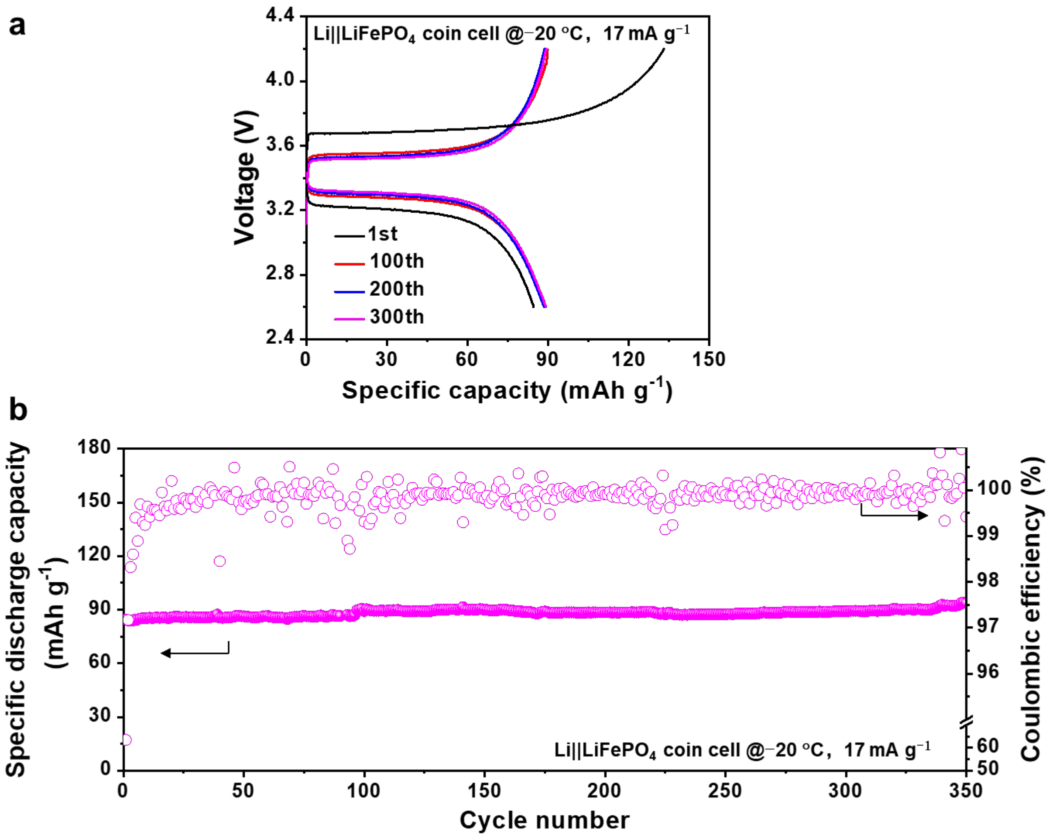

Supplementary Fig. 19 Electrochemical energy storage performances of the polymer electrolyte in Li||$LiFePO_4$ coin cell at –20 °C and 17 mA g^{-1}: **a**. typical charge-discharge profiles, **b**. cycling performances.

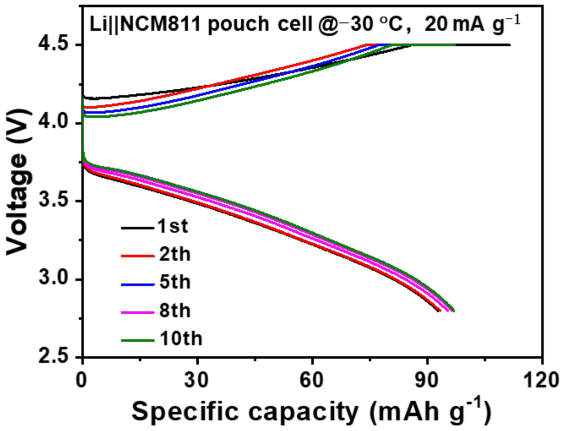

Supplementary Fig. 20 Typical charge-discharge voltage profiles of the polymer electrolyte in Li||NCM811 pouch cell at −30 °C.

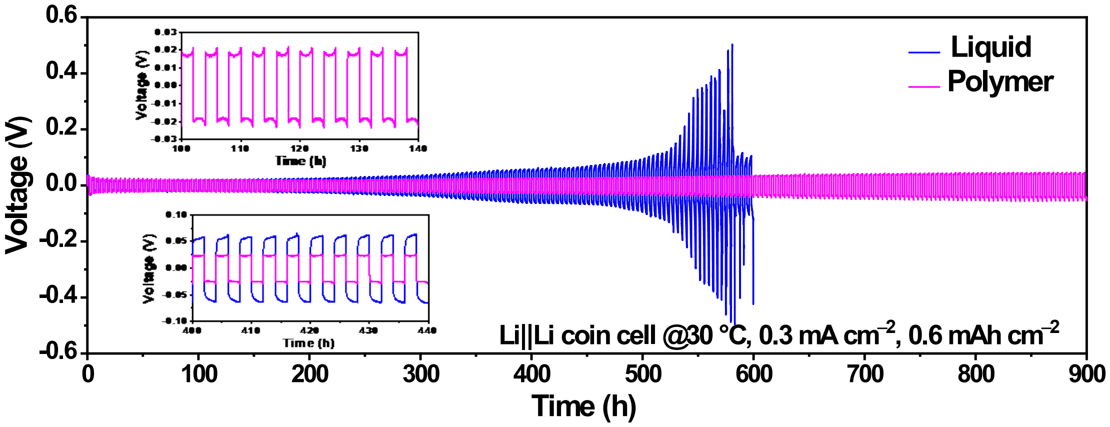

Supplementary Fig. 21 Galvanostatic cycling of the polymer electrolyte in symmetric Li||Li coin cell with 50 μm Li anode at 30 °C and 0.3 mA cm^{-2}; data for the liquid electrolyte are plotted for comparison.

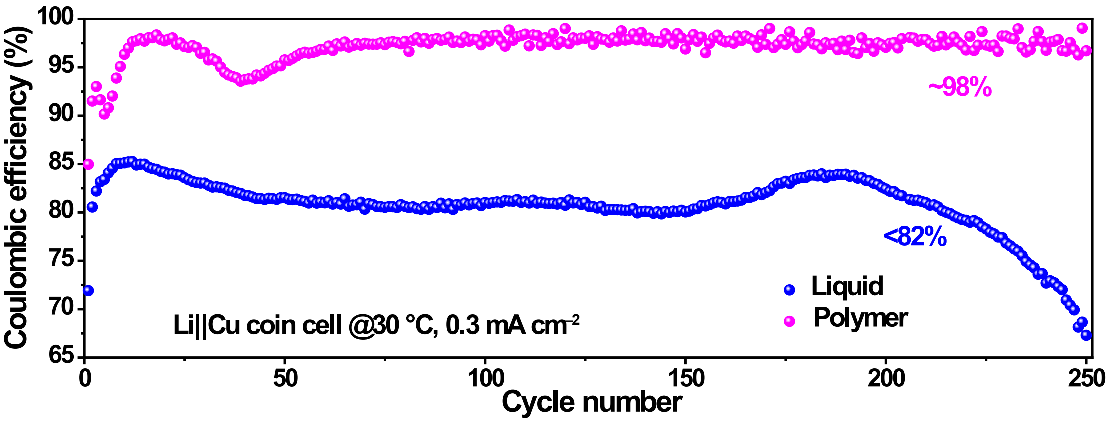

Supplementary Fig. 22 Coulombic efficiency of the polymer electrolyte in Li||Cu coin cell with 50 μm Li anode at 30 °C and 0.3 mA cm^{-2}; data for the liquid electrolyte are plotted for comparison.

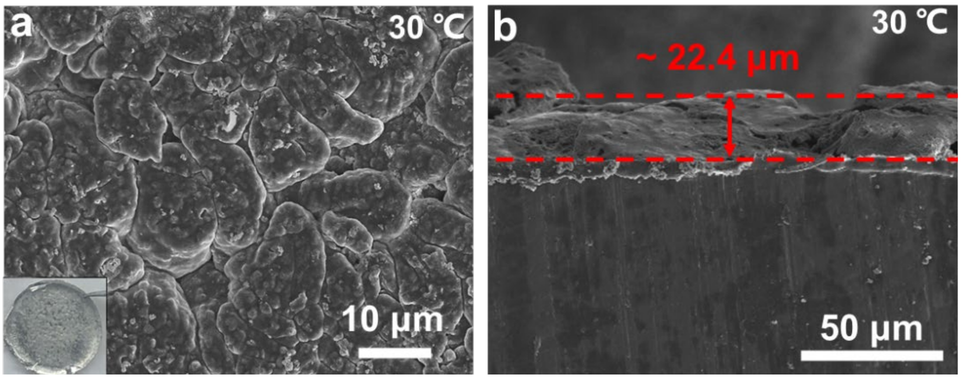

Supplementary Fig. 23 Morphologies of Li deposited at 30 °C: **a**. surface, **b**. cross-section. The deposited Li was disassembled from the Li||Li coin cells after 100 cycles at 30 °C and 0.2 mA cm^{-2}.

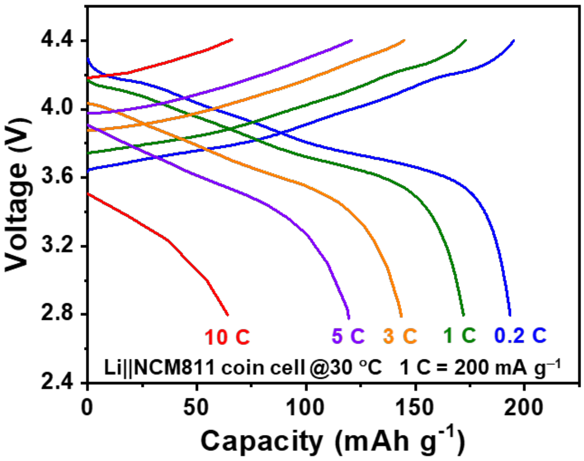

Supplementary Fig. 24 Charge-discharge voltage profiles of the liquid electrolyte in Li||NCM811 coin cell at different rates (30 °C). The second cycle at each rate are plotted.

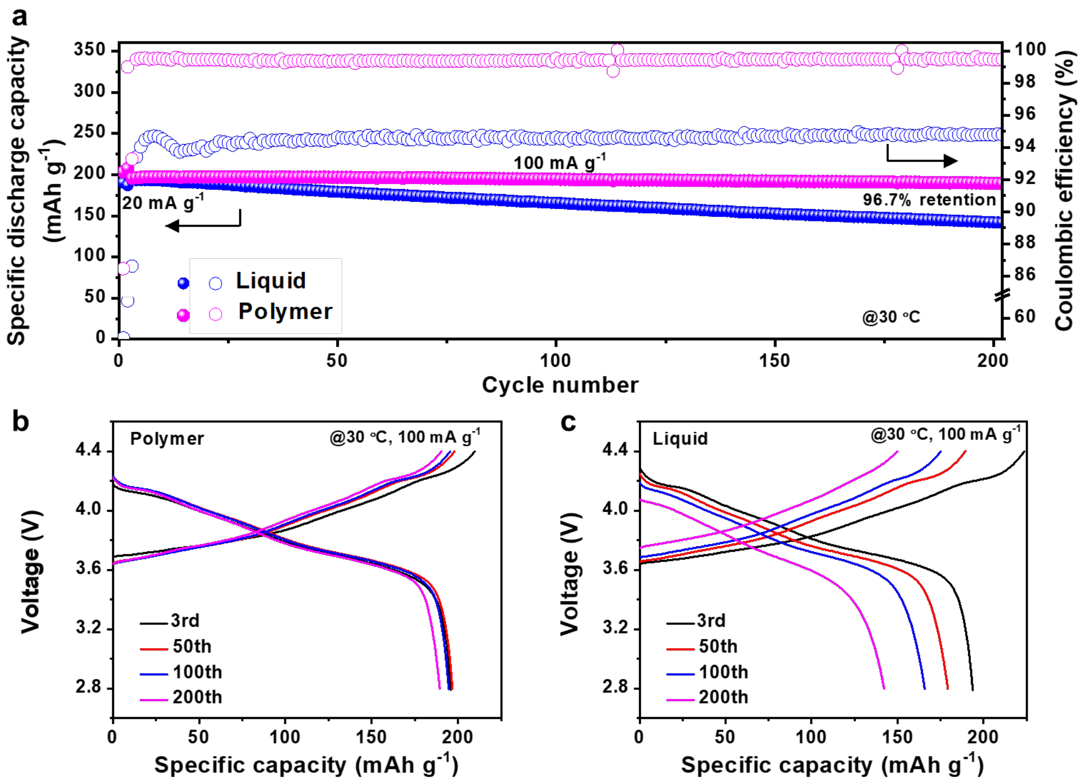

Supplementary Fig. 25 Battery performances of both electrolytes in Li||NCM811 coin cells at 30 °C: **a**. Cycling performances, **b**,**c**. corresponding charge-discharge profiles of the polymer electrolyte (**b**), and the liquid electrolyte (**c**). The first two formation cycles were carried out at a specific current of 20 mA g^{-1}, and the specific current of long-term cycling was set at 100 mA g^{-1}.

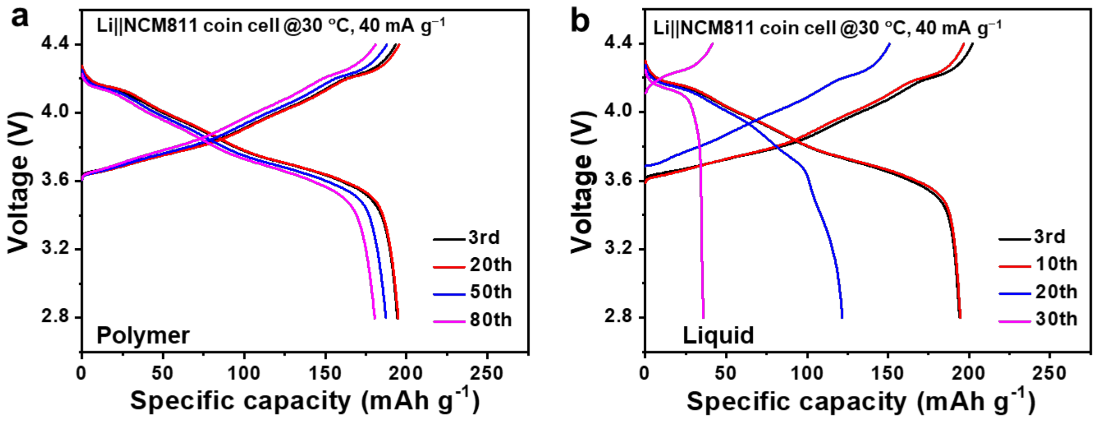

Supplementary Fig. 26 Charge-discharge voltage profiles of both electrolytes in Li||NCM811 coin cells at 30 °C and 40 mA g^{-1} under practical conditions (cathode loading of ~2.5 mAh cm^{-2}, negative to positive (N/P) ratio of ~3.86, electrolyte to capacity (E/C) ratio of ~5 g $(Ah)^{-1}$): **a**. Polymer electrolyte, **b**. liquid electrolyte.

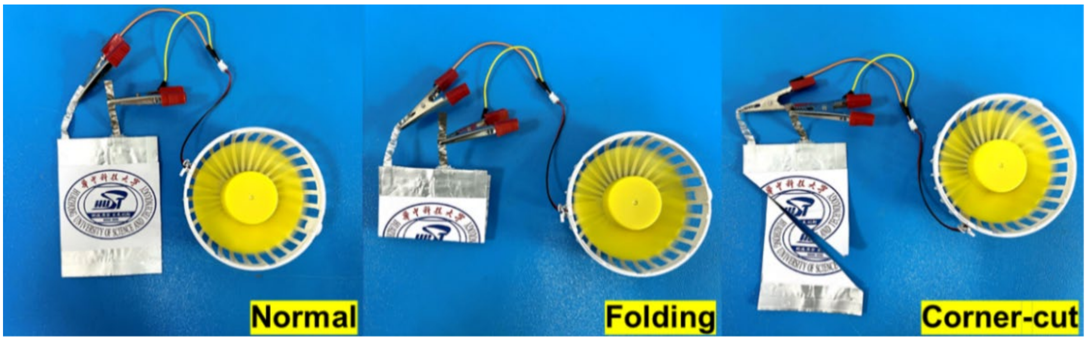

Supplementary Fig. 27 Li||NCM811 pouch cells using the polymer electrolyte are powering an electric fan under normal, folding, and corner-cut conditions.

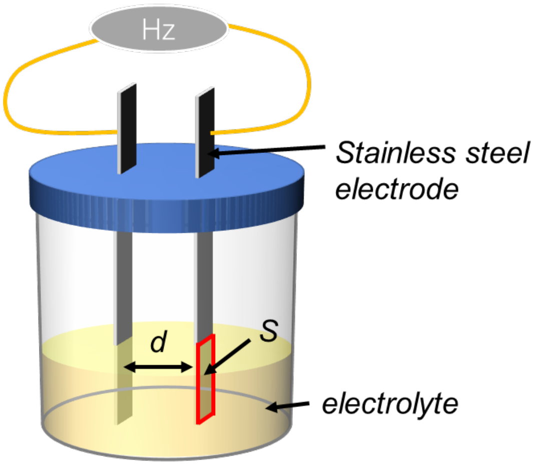

Supplementary Fig. 28 Schematic illustration of electrochemical impedance spectroscopy (EIS) measurement setup.

Supplementary Table 1 Gel permeation chromatography (GPC) results of electrolytes prepared at different TXE and FDMA ratios, where M_n is the number-average molecular weight, M_w is the weight-average molecular weight, M_P is the molecular weight of the highest peak, and PDI is the polydispersity.

Samples	M_n (Daltons)	M_w (Daltons)	M_P (Daltons)	PDI
5:1	16334	31011	31220	1.90
5:2	15804	31239	32692	1.98
5:3	13778	28987	30262	2.10
5:5	12295	29477	30863	2.39
3:5	11587	27898	28780	2.41

Supplementary Table 2 Cell performances of some typical polymer-based electrolytes. For the cells discussed in this table, a Li metal electrode thickness >250 μm and electrolyte flooded conditions were used. In the "Cathodes" column, the date in brackets is the wt.% of active material in the positive electrode and the electrode mass loading.

Electrolytes	Ionic conductivity	Transference number	Operation temperature	Cathodes (wt.% of active material, mass loading)	Cut-off Voltage	Performance	Ref.
Poly(VC)	3.03 mS cm^{-1} (30 °C)	0.71	25 °C	$LiNi_{0.8}Co_{0.1}Mn_{0.1}O_2$ (80%, 4.5 mg cm^{-2})	4.3 V	~150 mAh g^{-1}, 100 cycles, 93% retention, 90 mA g^{-1}	1
Poly(DOL)	>1 mS cm^{-1} (30 °C)	--	25 °C	$LiNi_{0.6}Co_{0.2}Mn_{0.2}O_2$ (3.0 mAh cm^{-2})	4.3 V	~153 mAh g^{-1}, 60 cycles, 80% retention, 0.3 mA cm^{-2}	2
Poly(DOL)	>1 mS cm^{-1} (30 °C)	--	25 °C	$LiFePO_4$ (84wt.%, 5 mg cm^{-2})	4.0 V	~95 mAh g^{-1}, 700 cycles	3
Poly(DOL)	1.163 mS cm^{-1} (30 °C)	0.58	−20 °C	S (60wt.%, 4 mg cm^{-2})	2.8 V	~700 mAh g^{-1}, 60 cycles	4
Poly(DOL)	--	0.64	15 °C	$LiFePO_4$ (80wt.%, 2 mg cm^{-2})	4 V	118 mAh g^{-1}, 1200 cycles, 87.2% retention	5
Poly(DOL)	2.8 mS cm^{-1} (30 °C)	0.61	−20 °C	$LiNi_{0.8}Co_{0.1}Mn_{0.1}O_2$ (80wt.%, 1.5 mg cm^{-2})	4.3 V	109 mAh g^{-1}, 100 cycles 18 mA g^{-1}	6
Poly(TXE)	0.114 S cm^{-1} (30 °C)	--	−10 °C	$LiCoO_2$ (80wt.%, 1.3 mg cm^{-2})	4.3 V	~158 mAh g^{-1} 200 cycles, 88% retention	7
PTFEMA	0.82 S cm^{-1} (25 °C)	0.24	0 °C	$LiFePO_4$ (80wt.%, 1.5 mg cm^{-2})	3.9 V	~86 mAh g^{-1}, 1 cycle, 17 mA g^{-1}	8
Poly(VEC)	2.1 mS cm^{-1} (25 °C)	0.40	−15 °C	$LiFePO_4$ (80wt.%, 3 mg cm^{-2})	4.0 V	~100 mAh g^{-1}, 1 cycle, 17 mA g^{-1}	9
Poly(TEGDA)	1.02 mS cm^{-1} (30 °C)	0.65	−10 °C	$LiFePO_4$ (80wt.%)	4 V	114 mAh g^{-1}, 50 cycles	10
Poly(TXE)	2.5 mS cm^{-1} (30 °C)	0.80	−20/−30 °C	$LiFePO_4$ (85wt.%, 3 mg cm^{-2})	4 V	~95 mAh g^{-1}, 350 cycles, −20 °C, 20 mA g^{-1}	This work
				$LiNi_{0.8}Co_{0.1}Mn_{0.1}O_2$ (85wt.%, 3 mg cm^{-2})	4.5 V	~150 mAh g^{-1}, 200 cycles, −20 °C ~92 mAh g^{-1}, >10 cycles, −30 °C, 20 mA g^{-1}	

Supplementary Note 1

As shown in Supplementary Fig. 2, LiDFOB firstly decomposes into LiF, BF_3 and lithium bis(oxalato)borate (LiBOB) on the surface of lithium metal with the aid of alkaline lithium.[11,12] BF_3 is known to be a strong Lewis acid, and also a critical initiator for polymerization,[13] which enables the polymerization to proceed smoothly. Namely, in the precursor solution, BF_3 combines with trace water to form $H^+(HOBF_3)^-$, inducing TXE monomers to convert into reactive oxonium ions via fast protonation, and the repetitious interposition of TXE monomers into oxonium ions, which leads to the polymer chain growth. As the polymer grows to a certain degree, trace H_2O attacks oxonium ions and terminates the current chain growth with a nucleophilic substitution. High–molecular weight linear-chain polymer polyoxymethylene (POM) is then acquired. Consequently, a homogeneous polymer-based electrolyte is eventually established via the combination of the polymer framework (POM) with FDMA.

As shown in Supplementary Fig. 3, after the polymerization, the doublet absorption peaks attributed to the anti-symmetric vibration mode of -O-C-O- group (TXE) shift from 1159.84 and 1082.2 cm^{-1} to 1174.71 and 1078.94 cm^{-1}, respectively. In addition, the missing of C-H out of the plane vibration at 928.56 cm^{-1} and the shift of absorption peaks from 2851.9 and 2922.6 cm^{-1} to 2850.7 and 2939.1 cm^{-1}, respectively, which are resulted from the $-CH_2-$ stretching mode, also validate that TXE is successfully polymerized to POM.

The result of the Raman characterization is consistent with the finding of the FTIR spectra. After the polymerization, the signal (973 cm^{-1}) belonging to the C-O-C ring

stretching is missing. In addition, new peak (920 cm^{-1}) associated with the C-O-C symmetric stretching appears. And peaks belonging to the CH_2 symmetric stretching shift from 2802, 2884, 2960 and 3032 cm^{-1} to 2822, 2929, 3005 and 3051 cm^{-1}, respectively, indicating the successful polymerization of TXE.

After the polymerization reaction, new hydrogen and carbon peaks were observed, which are in accordance with the structure of POM (Supplementary Fig. 5), demonstrating that TXE is completely polymerized and transformed to POM. Additionally, the polymer shows an average molecular weight of ~13778 (Supplementary Fig. 6 and Supplementary Table 1), indicating the successful polymerization of TXE (with a molecular weight of ~90.08).

As shown in Supplementary Fig. 7, originally flowable liquid solution of TXE-FDMA-FEC-LiDFOB precursor turns into a solid-like electrolyte with immovable characteristics. More importantly, the electrolyte remains solid even at an elevated temperature of 100 °C. These results demonstrate that polymerization has taken place in the electrolyte.

Supplementary Note 2

To achieve satisfactory conductivity values, liquid plasticizers (*e.g.* FDMA) are inevitably required. When the mass ratios of TXE and FDMA are 5:1 and 5:2, they have relatively low ionic conductivities at room temperature. With increasing ratio of FDMA, the ionic conductivity of the electrolyte rapidly increases (Supplementary Fig. 8).

As listed in Supplementary Table 1, M_n of polymerized electrolytes generally

decreases with the increment of the FDMA content. The higher content of FDMA retards the reaction rate, causing polymer chains to grow slowly, which would lower the polymer molecular weight. At the TXE-FDMA mass ratios of 5:5 and 3:5, the electrolytes contain much low-molecule polymers. High content low-molecule polymers and solvents not only cause poor thermal/mechanical stability and safety hazards such as fire and explosion during thermal runaway, but also greatly deteriorates the electrode/electrolyte interfaces.

In view of the balance between the ionic conductivity and the thermal/mechanical/(electro)chemical stability, the electrolyte with the TXE-FDMA ratio of 5:3 exhibits a suitable molecular weight (M_n = 13778), a relatively narrow polydispersity (2.10) and an acceptable ionic conductivity. Hence, we finally choose the polymer electrolyte with the TXE and FDMA ratio of 5:3 as the electrolyte for further investigations.

Supplementary Note 3

According to the cyclic voltammetry (CV) tests (Supplementary Fig. 12), the reference liquid electrolyte exhibits two additional reduction peaks at ~1.4 V and ~0.5 V, which represent the reduction of $LiPF_6$ salts and carbonate solvents, respectively. In contrast, the polymer electrolyte demonstrates much slighter reduction peaks, indicating no obvious side-reaction between the polymer electrolyte and LMA.

Supplementary Note 4

As shown in Supplementary Fig. 21, in the reference liquid electrolyte system, an

obvious but gradual increase in voltage is observed after 250 h at room temperature; in comparison, the cell with the polymer electrolyte exhibits very stable cycling for more than 900 h.

As shown in Supplementary Fig. 22, in the reference liquid electrolyte system, the Li Coulombic efficiency is lower than 82% and fades quite quickly at room temperature; in sharp contrast, the Li||Cu cell with the polymer electrolyte shows stable cycling with a much higher Coulombic efficiency of ~98%.

At room temperature, large and more compact granular Li particles with a size of ~10 μm are observed in the polymer system, as shown in the SEM image (Supplementary Fig. 23a); the deposited layer is only ~22.4 μm in thickness (Supplementary Fig. 23b), indicating minimized Li loss and volumetric expansion.

Supplementary references

1. Ma, Q., *et al.* Formulating the Electrolyte Towards High-Energy and Safe Rechargeable Lithium-Metal Batteries. *Angew.Chem. Int. Ed.* **60**, 16554-16560 (2021).
2. Zhao, C.Z., *et al.* Rechargeable Lithium Metal Batteries with an In-Built Solid-State Polymer Electrolyte and a High Voltage/Loading Ni-Rich Layered Cathode. *Adv. Mater.* **32**, 1905629 (2020).
3. Zhao, Q., Liu, X., Stalin, S., Khan, K. & Archer, L.A. Solid-state polymer electrolytes with in-built fast interfacial transport for secondary lithium batteries. *Nat. Energy* **4**, 365-373 (2019).
4. Xiang, J., *et al.* A flame-retardant polymer electrolyte for high performance lithium metal batteries with an expanded operation temperature. *Energy Environ. Sci.* **14**, 3510-3521 (2021).
5. Chen, Y., Huo, F., Chen, S., Cai, W. & Zhang, S. In-Built Quasi-Solid-State Poly-Ether Electrolytes Enabling Stable Cycling of High-Voltage and Wide-Temperature Li Metal Batteries. *Adv. Funct. Mater.* **31**, 2102347 (2021).
6. Yu, J., *et al.* In Situ Fabricated Quasi-Solid Polymer Electrolyte for High-Energy-Density Lithium Metal Battery Capable of Subzero Operation. *Adv. Energy Mater.* **31**, 2102932 (2021).
7. Wu, H., *et al.* LiDFOB Initiated In Situ Polymerization of Novel Eutectic Solution Enables Room-Temperature Solid Lithium Metal Batteries. *Adv. Sci.* **7**, 2003370 (2020).
8. Yu, L., *et al.* Monolithic Task-Specific Ionogel Electrolyte Membrane Enables High-Performance Solid-State Lithium-Metal Batteries in Wide Temperature Range. *Advan. Funct. Mater.* **32**, 2110653 (2021).
9. Lin, Z., *et al.* A wide-temperature superior ionic conductive polymer electrolyte for lithium metal battery. *Nano Energy* **73**, 104786 (2020).
10. Li, Z., *et al.* Nonflammable quasi-solid electrolyte for energy-dense and long-cycling lithium metal batteries with high-voltage Ni-rich layered cathodes. *Energy Storage Mater.* **47**, 542-550 (2022).
11. Zhou, L., Li, W., Xu, M. & Lucht, B. Investigation of the Disproportionation Reactions and Equilibrium of Lithium Difluoro(Oxalato) Borate (LiDFOB). *Solid-State Lett.* **14**, A161 (2011).
12. Liu, Q., *et al.* Long-cycling and safe lithium metal batteries enabled by the synergetic strategy of ex situ anodic pretreatment and an in-built gel polymer electrolyte. *J. Mater. Chem. A* **8**, 7197-7204 (2020).
13. Cui, Y., *et al.* High Performance Solid Polymer Electrolytes for Rechargeable Batteries: A Self-Catalyzed Strategy toward Facile Synthesis. *Adv. Sci.* **4**, 1700174 (2017).
