# Supplementary material for: Tailoring polymer electrolyte ionic conductivity for production of low- temperature operating quasi-all-solid-state lithium metal batteries
Source: Nat Commun. 2023 Jan 30;14:482. doi: 10.1038/s41467-023-35857-x (PMC9886912; doi:10.1038/s41467-023-35857-x)
Supplement: Supplementary file 3 — Description of additional Supplementary File [file 41467_2023_35857_MOESM3_ESM.pdf]

<u>**Descriptions of additional Supplementary Files**</u>

Supplementary Movie 1: A Li||NCM811 pouch cell using the designed polymer electrolyte was powering an electric fan at –48.2 °C.
